# Are DMARDs enough to prevent surgery in rheumatoid hands?

**DOI:** 10.1186/1753-6561-9-S3-A91

**Published:** 2015-05-19

**Authors:** Yeap Swan Sim

**Affiliations:** 1 Department of Medicine, Subang Jaya Medical Center, Selangor, 47500, Malaysia

## The hands are commonly involved in RA

Rheumatoid arthritis (RA) is a chronic, auto-immune disorder characterised by inflammation primarily in the joints. The small joints in the hands are typically involved in RA, with both the 1987 American Rheumatism Association revised criteria for RA classification including “arthritis of hand joints” as one of the criterion [1] and the 2010 RA classification criteria awarding more points for small joint involvement (i.e. themetacarpophalangeal joints, proximal interphalangeal joints, second to fifth metatarsophalangeal joints, thumb interphalangeal joints and wrists) [2].

RA is the most common of the inflammatory arthritides; a study in the UK found the prevalence of RA to be 1.16% in women and 0.44% in men [3]. In Asia, the prevalence of RA varies from country to country. In rural areas, the prevalence of RA is 0.12% in Thailand, 0.2% in Indonesia, 0.26% in Taiwan, 0.3% in Malaysia, 0.34% in China, 0.55% in India and 0.7% in Bangladesh [4].

## Pathogenesis of joint destruction

Although a specific antigen that triggers off the immune response in RA has not been convincingly identified, cell-mediated immune responses (especially from the T cells) have been shown to be responsible for RA joint inflammation. In an susceptible host (e.g. a person with the appropriate genetic background), the T cell response results in the elaboration of T cell cytokines, with resultant recruitment of inflammatory cells, including neutrophils, macrophages, B cells and memory T cells. A target of the inflammatory cells is the synovium which becomes markedly hyperplastic and infiltrated with mononuclearcells. One feature of RA that distinguishes it from other inflammatory arthropathies is the propensity for the synovium to become locally invasive at the synovial interface with cartilage and bone. This destructive mass is called “pannus” and is responsible for the characteristic marginal erosions observed in RA [5]. Cartilage is further destroyed in RA by the catabolic effects of cytokines such as IL-1, IL-6 and tumour necrosis factor-alpha (TNF-α) and the production of metalloproteinases, which can degrade extra-cellular matrix/cartilage, causing more joint damage[5].

Apart from synovitis, inflammation of the adjacent intertrabecular space (osteitis) also correlates with the development of radiographic bone erosions.Articular bone erosion represents localized bone loss (osteolysis),which results from an imbalance in which bone resorption by osteoclasts is favoured over bone formation by osteoblasts [6]. In RA, TNF-α and receptor activated nuclear factor κ ligand (RANKL) are key cytokines mediating the activation of the osteoclasts [7].

Studies have shown that erosive disease develops early in the course of RA in the majority of patients. The rate of progression is fastest in the first 2 years [8]. Longitudinal studies have shown that after 2 years approximately 36% of patients have erosions, 47% by 5 years [9] and 63% by 8 years [10]. If both joint space narrowing and erosions are considered, up to 70% of patients will show radiographic damage after 3 years [11].

## Does treatment prevent erosions?

Treatment for RA must involve suppression of the immune-mediated inflammatory response, which requires the use ofdisease-modifying anti-rheumatic drugs (DMARDs). Methotrexate (MTX) is the anchor synthetic DMARD.Good control of inflammation/remission (no joint swelling or tenderness, normal ESR/CRP) will reduce the rate of progression of erosions [12] and hence joint damage. Compared to placebo, MTX is effective in reducing joint pain and swelling, improving function and reducing the ESR. However, its efficacy at inducing disease remission is limited (only in approximately 25% of patients) andhence less efficacious at reducing radiographic progression [13]. Biologics are monoclonal antibodies produced against various inflammatory cytokines such as TNF-α or IL-6. Compared to MTX, biologics are very much more effective atinducing disease remission and hence reducing the progression of joint damage as assessed by radiographic change scores (e.g. [13-15]. Unfortunately, a major barrier to the widespread use of biologics is their high cost. In addition, approximately 20% of patients do not achieve adequate disease suppression with biologic treatment [16]. So, despite the availability of treatment, many patients with RA remain undertreatedand thus have continuing joint destruction. Nonetheless, since the mid-1990s, studies have shown that the amount of hand surgery in RA patients has been slowly declining, postulated to improvements in medical therapy [17,18].

## Conclusion

Adequate and early treatmentof RA does reduce the rate of radiographic progression and hence joint damage. Although the drugs are available, the most effective drugs are not widely used due to cost issues. Thus, RA hand surgery will still be needed in the foreseeable future.
